# Managing first-line failure

**DOI:** 10.7448/IAS.17.4.19489

**Published:** 2014-11-02

**Authors:** David A Cooper

**Affiliations:** Kirby Institute, University of New South Wales, Sydney, Australia

## Abstract

WHO standard of care for failure of a first regimen, usually 2N(t)RTI's and an NNRTI, consists of a ritonavir-boosted protease inhibitor with a change in N(t)RTI's. Until recently, there was no evidence to support these recommendations which were based on expert opinion. Two large randomized clinical trials, SECOND LINE and EARNEST both showed excellent response rates (>80%) for the WHO standard of care and indicated that a novel regimen of a boosted protease inhibitor with an integrase inhibitor had equal efficacy with no difference in toxicity. In EARNEST, a third arm consisting of induction with the combined protease and integrase inhibitor followed by protease inhibitor monotherapy maintenance was inferior and led to substantial (20%) protease inhibitor resistance. These studies confirm the validity of the current recommendations of WHO and point to a novel public health approach of using two new classes for second line when standard first-line therapy has failed, which avoids resistance genotyping. Notwithstanding, adherence must be stressed in those failing first-line treatments. Protease inhibitor monotherapy is not suitable for a public health approach in low- and middle-income countries.

